# Appetite Suppression and Antiobesity Effect of a Botanical Composition Composed of* Morus alba*,* Yerba mate*, and* Magnolia officinalis*

**DOI:** 10.1155/2016/4670818

**Published:** 2016-09-06

**Authors:** Mesfin Yimam, Ping Jiao, Mei Hong, Lidia Brownell, Young-Chul Lee, Eu-Jin Hyun, Hyun-Jin Kim, Tae-Woo Kim, Jeong-Bum Nam, Mi-Ran Kim, Qi Jia

**Affiliations:** ^1^Unigen Inc., 3005 1st Avenue Seattle, WA 98121, USA; ^2^Unigen Inc., No. 450-86, Maebong-Ro, Dongnam-Gu, Cheonan-Si, Chungnam 330-863, Republic of Korea

## Abstract

*Background*. Obesity and its comorbidities continue to challenge the world at an alarming rate. Although the long term solution lies on lifestyle changes in the form of dieting and exercising, drug, medical food, or dietary supplement interventions are required for those who are already obese. Here we describe a standardized blend composed of extracts from three medicinal plants:* Morus alba*,* Yerba mate*, and* Magnolia officinalis* for appetite suppression and metabolic disorders management.* Method*. Extracts were standardized to yield a composition designated as UP601. Appetite suppression activity was tested in acute feed intake rat model. Efficacy was evaluated in C57BL/6J mouse models treated with oral doses of 1.3 g/kg/day for 7 weeks. Orlistat at 40 mg/kg/day was used as a positive control. Body compositions of mice were assessed using a dual energy X-ray absorptiometry (DEXA). ELISA was done for insulin, leptin, and ghrelin level quantitation. Nonalcoholic steatohepatitis (NASH) scoring was conducted.* Results*. Marked acute hypophagia with 81.8, 75.3, 43.9, and 30.9% reductions in food intake at 2, 4, 6, and 24 hours were observed for UP601. Decreases in body weight gain (21.5% compared to the HFD at weeks 7 and 8.2% compared to baseline) and calorie intake (40.5% for the first week) were observed. 75.9% and 46.8% reductions in insulin and leptin, respectively, 4.2-fold increase in ghrelin level, and reductions of 18.6% in cholesterol and 59% in low-density lipoprotein were documented. A percentage body fat of 18.9%, 47.8%, 46.1%, and 30.4% was found for mice treated with normal control, HFD, Orlistat, and UP601, respectively. 59.3% less mesenteric fat pad and improved NASH scores were observed for UP601.* Conclusion*. UP601, a standardized botanical composition from* Morus alba*,* Yerba mate*, and* Magnolia officinalis* could be used as a natural alternative for appetite suppression, maintaining healthy body weight and metabolism management.

## 1. Introduction

Obesity and its comorbidities continue to challenge the world at an alarming rate. In recent years it has been reported that obesity and its metabolic complications will cause both substantial socioeconomic and physical burdens on society [1]. It is widely recognized as the largest and fastest growing public health problem worldwide affecting both adults and children. For example, recent pooled data from 186 countries for the duration 1975–2014 showed that obese people worldwide had risen from 105 million in 1975 to 641 million in 2014 [2]. The research also found that more obese men and women now live in China (43.2 million men and 46.4 million women) and the USA (41.7 million obese men and 46.1 million obese women) than in any other country. It is often caused by an imbalance between energy intake and expenditure which mainly complicated with a much more sedentary lifestyle associated with easy access of palatable energy dense diet rich in fat, sugar, and salt. If untreated, it can lead to diabetes, hypertension, stroke, dyslipidemia, and other more deleterious complications [3].

Despite the fact that the long-term solution for obesity lies on lifestyle changes in the form of dieting and exercise, interventions are required for those who already are obese. The conventional pharmaceutical drugs are considered the primary choices and generally effective to carve the excess weight. However, for most, besides being too expensive, the long-term uses of these drugs are marred by their severe adverse toxicities. In fact, the principal reason for drug withdrawals has been due to concerns over safety rather than the effect on body weight. For example, the withdrawal of Acomplia (rimonabant), appetite suppressant acting as cannabinoid (CB1) receptor antagonist due to the risk of psychiatric side effects, including depression, sleep disorders, anxiety, and aggression in 2008, and the suspension of the use of another appetite suppressant, sibutramine, a monoamine reuptake inhibitor as a result of increased risk of serious, nonfatal cardiovascular events such as stroke and heart attack in 2010, are some of the disappointing trends of obesity drug development [4]. As a result, there remains significant unmet need for the discovery of safer weight management alternatives delivering superior efficacy. In this regard, herbs with a long history of use are less likely to produce severe toxicity and might be effective in reducing appetite and promoting significant weight loss. Therefore, a natural product based intervention could be an inexpensive and relatively safer substitute to combat obesity and aid healthy weight management [5]. Here, we have documented data for a novel standardized composition designated as UP601 comprised of extracts from* Magnolia officinalis*,* Morus alba*, and* Yerba mate*.

Augmented preclinical and some preliminary clinical efficacy of* Magnolia officinalis*,* Morus alba*, and* Yerba mate* extracts in managing healthy blood glucose levels and improving metabolic disorders have been reported separately.

For instance, (1) improving insulin sensitivity through the activation of PPAR*γ* as a ligand [6] and relieving anxiety-related disorders such as anxiety neurosis [7] by acting as GABA-A receptor agonists [8] or through an indirect cholinergic activity such as inhibition of histaminergic neurons linked to cholinergic neurons [9, 10] have been reported for* Magnolia* extracts or its main active components, magnolol and honokiol.

(2) Likewise, multiple animal studies have shown that* Yerba mate* extract alleviates weight gain and improves plasma glucose and lipid profiles. A reduction in preadipocyte differentiation, decrease in adipocyte lipid accumulation, a decrease in food intake, a decrease in body weight, reductions in serum cholesterol, serum triglycerides, and blood glucose concentrations [11–14], reduction in insulin and leptin levels [15], and adipogenesis gene regulation [15] of* Yerba mate* were reported.

(3) Traditionally,* Morus alba* has been used as a herbal remedy for various types of diseases including metabolic disorders like diabetes and obesity. Significant amount of reports have been documented to validate its value* in vivo* and* in vitro*. In animal studies, long-term administration of* Morus* extract was found to decreases body weight and adiposity and regulates hepatic lipid accumulation in DIO mice [16]. Similarly, improved insulin resistance [17], decreased expression of white muscle adipocytokines in db/db mice [18], and decreased blood glucose level in alloxan diabetic mice [19] were reported from* in vivo* studies as a result of oral administration of extract of* Morus*.* In vitro*, increased adiponectin from murine adipocyte [20], increased glucose uptake and GLUT4 translocation in rat adipocytes [20], and inhibition of *α*-glucosidase activity [21] were reported.

Given these historical usage and contemporary pharmacological significance, formulating these well-known plant materials for alternative botanical products may have a merit of use in appetite suppression and managing healthy body weight. As described above, the use of extracts from* Yerba mate*,* Morus*, and* Magnolia* for blood glucose and/or lipid management has been reported separately. To the best of our knowledge, this is the first report of its kind to put extracts from all these three traditionally well-attested plants together for appetite suppression and metabolic disorder related evaluations. Prior to the present study, as a hit from* in vivo* efficacy screening, each plant material was tested in house individually at multiple dosages and formulated for a potential viable composition with a better efficacy. Each component was also compared against the composition as they appear in the composition leading us to believe that putting these plant materials together at this specific ratio may have some merit for a better outcome (USPT: 20140004215). The current study was designed to assess efficacy of a specific blend of these three extracts to suppress appetite and to ameliorate a number of obesity-related phenotypic and biochemical markers in a HFD-induced mouse model of obesity.

## 2. Materials and Method

### 2.1. Material Preparation

Detailed procedures of composition matter preparations have been described on US patent application entitled “Compositions and Methods for Managing Weight” with publication number: 20140004215.

UP601 is a proprietary blend of three standardized extracts from* Magnolia officinalis* stem bark,* Morus alba* root bark, and* Ilex paraguariensis* leaf with not less than 7% magnolol and honokiol, 2% caffeine, and 1% total bioflavonoids including kuwanon G and albanin G and morusin.

*Morus alba* root bark extract was produced by 70% ethanol extraction of the ground root bark powder with no less than 10% total bioflavonoids including kuwanon G and albanin G and morusin.* Magnolia officinalis* stem bark was extracted by a supercritical fluid and further crystalized to give a mixture of magnolol and honokiol with a content higher than 95%.* Ilex paraguariensis* leaf was extracted with water to give* Yerba mate* extract containing not less than 4% caffeine. The dried powders of* Magnolia officinalis* stem bark extract,* Morus Alba* root bark extract, and* Ilex paraguariensis* leaves extracts were mixed at a proprietary ratio to produce the standardized UP601 composition.

### 2.2. Acute Feed Intake Study

Male Sprague-Dawley rats (Harlan), weighing 230 to 250 g, were used for the acute food intake study. Upon arrival, animals were acclimated in the holding room controlled for temperature and light. The light and dark cycles were reversed (lights off at 10:00 AM–10:00 PM) to acquaint the nocturnal circadian rhythmicity. All food intake studies were conducted during the dark phase. During these periods, rats were allowed* ad libitum* access for regular rodent pellets and water.

On the day of testing, 16-hour fasted animals (*N* = 8 per group), single housed in cages with grid floors, were allowed to acclimate to these cages for a 90-minute period, with free access to water and no food. At 10:00 AM, vehicle (0.5% CMC) or UP601 (230 and 350 mg/kg) via oral gavage was administered to rats. Thirty minutes after administration (10:30 AM), lights were turned off, and animals were allowed access to a highly palatable 45% Kcal high-fat diet (TD06415, Harlan). Food consumption was then monitored at 1, 2, 4, 6, 8, 10, and 24 hours after food exposure.

### 2.3. DIO Induction and Intervention

A high-fat diet (HFD) induced obese mice animal model was developed and utilized. Methodologies of obesity induction have been described before [22]. In brief, obesity was induced in male C57BL/6J mice (5 weeks old, 18~24 g average body weight, obtained from Korea Research Institute of Bioscience & Biotechnology) when fed a high-fat diet of 60% Kcal fat (Research diet D12492, Doo Yeol Biotech) for 8 weeks. During induction period, body weights were taken once per a week for 8 weeks where at the end a 38.8% increase in body weight gain was observed. Once induction was confirmed, mice were randomized into four groups of (1) normal control + vehicle (*N* = 10, normal diet, research diet D12450B), (2) HFD + vehicle (*N* = 10), (3) HFD + Orlistat (*N* = 10, 40 mg/kg), and (4) HFD + UP601 (*N* = 10, 1.3 g/kg) and fractionated dosage administrations were initiated orally twice per day to give the full daily dosages for each group and sustained for 7 weeks. The vehicle treated animals received 0.15% xanthan gum + 0.5% Tween 80 only in both studies. The positive control Orlistat (Lipidown Cap 120 mg, Lot #12003, Hanmi, Korea), N-Formyl-L-leucine (1S)-1-[[(2S,3S)-3-hexyl-4-oxo-2-oxetanyl]methyl]dodecyl ester, with a trade name of Xenical or Alli is a Food and Drug Administration approved human pancreatic lipase inhibitor which inhibits the absorption of approximately one-third of fat from ingested food that would ultimately result in weight reduction.

Water was provided* ad libitum*. Animals were maintained in a temperature and air flow controlled room (22.2°C and 10–15 filtered air changes per hour, resp.) on a 12-hour light-dark cycle with relative humidity of 50°C ± 10. Feed and water consumption was measured twice a week throughout treatment period in both studies. To better estimate calorie intake by mice, instead of 5 mice/cage, as of week 6, each mouse was placed in an individual cage for the whole treatment duration. All animal experiments were conducted according to institutional guidelines congruent with the guide for the care and use of laboratory animals under Institutional Animal Care and Use Committees (IACUC) Approval number UIK21407.

### 2.4. Body Composition Analysis Using DEXA

After 6 weeks of treatment, sedated mice were subjected to dual energy X-ray absorptiometry (DEXA, Lunar PIXImus, GE, USA) for body composition of fat and lean mass analysis. The system was calibrated according to manufacturer's instructions prior to the start of the experiment. Software integrated to the scan was used for data analysis. DEXA uses two separate times of low-dose X-ray exposure to read bone and soft tissue mass with a high degree of precision.

### 2.5. Blood Chemistry Analysis for Liver Function and Lipid Profiles

At necropsy day, mice were fasted for 16 hours and approximately 0.6~0.9 mL of blood sample was collected from abdominal vein. Samples were centrifuged and serum was transferred to Biotoxtech Co. Ltd., Republic of Korea, for liver function and lipid profiles analysis. Serum levels of alanine aminotransferase (ALT), aspartate aminotransferase (AST), total cholesterol (T-Chol), triglycerides (TG), LDL-cholesterol (LDL-C), HDL-cholesterol (HDL-C), and glucose were measured using Hitachi autoanalyzer (7180, Hitachi, Japan).

### 2.6. Necropsy and Tissue Collection for Histopathology

On the last day of the assay, all animals were exsanguinated and examined for gross pathology. Once the abdominal cavity was opened, organs were subjected to gross examination. Liver and visceral fat pads (epididymal, retroperitoneal, perirenal fat pad, and mesenteric fat) were collected and weighed individually for organ-to-body weight ratio determination and then specimens were fixed with 10% buffered neutral formalin, trimmed, processed, embedded in paraffin, sectioned, and stained with Hematoxylin & Eosin (H&E) for microscopic NASH (nonalcoholic steatohepatitis) score analysis according to modified scoring system method of Kleiner et al. [23].

### 2.7. Biomarkers

At the end of the 7th week of treatment, blood samples of approximately 0.6~0.9 mL were collected from abdominal artery of ether anesthetized mice after 16 hours of fasting. The blood sample was centrifuged at 2,000 ×g for 10 min at 4°C. After centrifugation, serum of each group was pooled, divided, and stored at −70°C until being analyzed. Enzyme-linked immunosorbent assay (ELISA) kits were used to determine the levels of leptin (#EZML-82K, Millipore Co., USA), active ghrelin (#EZRGRA-90K, Millipore Co., USA), and insulin (#EZRMI-13K, Millipore Co., USA). Measurements were performed using microplate leader (VictorTM X3, PerkinElmer Inc., USA) with software version of PerkinElmer 2030.

### 2.8. Statistical Analysis

All nondiscrete data from clinical chemistry, body weights, and food consumption were represented as mean ± S.D and were analyzed using SigmaPlot (version 11.0). Statistical significance between groups was calculated by means of single factor analysis of variance followed by a paired *t*-test. *P* values less than or equal to 0.05 (*P* ≤ 0.05) were considered as significant. When normality tests failed, data for nonparametric analysis were subjected to Mann-Whitney sum ranks for *t*-test and Kruskal-Wallis one way analysis of variance on ranks for ANOVA. Interpretations of the results were made based on findings from the in-life body weights and feed consumption data, DEXA scan, serum biomarkers, and NASH score.

## 3. Results

### 3.1. Acute Food Intake

**Figure 1:**
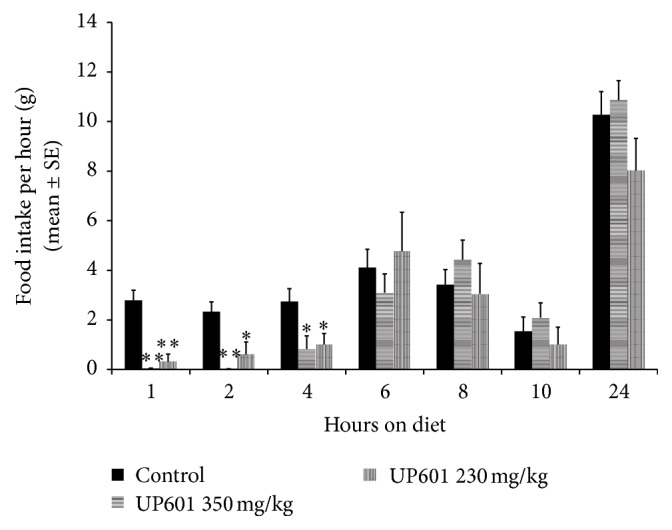
Hourly calorie intake by rats treated with UP601 at 230 and 350 mg/kg in the acute food intake study. ^*∗∗*^ *P* ≤ 0.0001; ^*∗*^ *P* ≤ 0.05.

**Figure 2:**
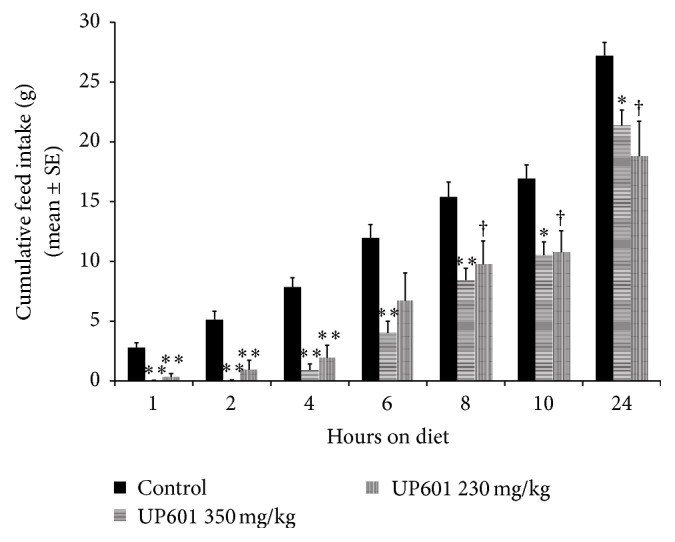
Cumulative food intake by rats treated with UP601 at 230 and 350 mg/kg in the acute food intake study. ^*∗∗*^ *P* ≤ 0.0001; ^*∗*^ *P* ≤ 0.001; ^†^ *P* ≤ 0.05.

Statistically significant reductions in acute food intake were observed for the first 4 hours after food provision for rats treated with UP601 (230 and 350 mg/kg) when consumption was monitored hourly. The mean ± SE values of food consumption were 0.04 ± 0.02, 0.03 ± 0.00, and 0.83 ± 0.53 grams for the 350 mg/kg UP601 and 0.32 ± 0.30, 0.61 ± 0.50, and 1.01 ± 0.44 grams of food for the 230 mg/kg UP601 treatment group at 1, 2, and 4 hours after food exposure (Figure 1). In the same duration, the control group consumed 2.79 ± 0.41, 2.33 ± 0.40, and 2.74 ± 0.52 grams of food. These reductions in percentages were 88.5, 73.8, and 63.1% for the 230 mg/kg UP601 and 98.6, 98.7, and 69.7% for the 350 mg/kg UP601 treatment groups compared to vehicle treated control. Similarly, the cumulative food intake data showed statistically significant reductions in food intake for 24 hours except for the 250 mg/kg UP601 group which did not show the significance at the 6-hour time point (Figure 2). The cumulative percent reductions were 81.8, 75.3, 43.9, and 30.9% for the 230 mg/kg UP601 and 98.6, 88.5, 66.6, and 21.4% for the 350 mg/kg UP601, respectively, compared to vehicle treated control.

### 3.2. Effect of UP601 on Body Weight in the DIO Model

**Figure 3:**
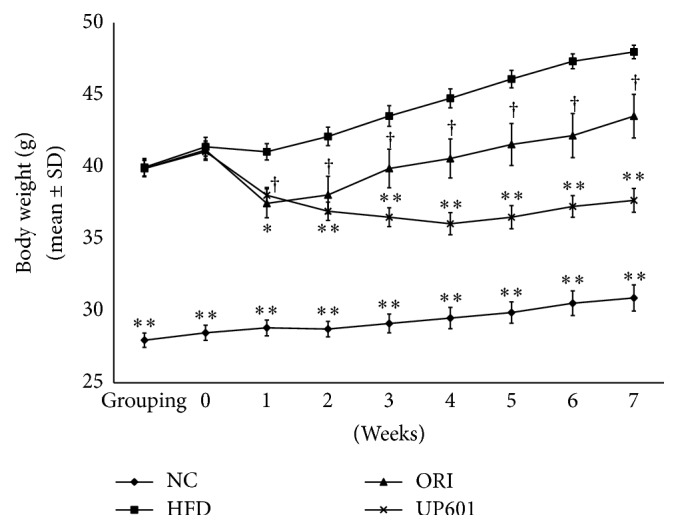
Body weight changes observed after 7 weeks of daily oral UP601 treatment in HFD fed obese C57BL/6J mice. ^*∗∗*^ *P* ≤ 0.0001; ^*∗*^ *P* ≤ 0.001; ^†^ *P* ≤ 0.05.

A progressive and stable body weight increase was observed when C57BL/6J mice (5 mice/cage) were provided with a 60% Kcal high-fat diet* ad libitum* for 6 weeks. Mice were transferred to a “minicage” that houses a single mouse per a cage as of week 6 for additional two weeks of induction and the duration of treatment on the same diet. After 8 weeks on the HFD, a 38.8% increase in body weight gain was observed and deemed mice were ready for randomization and treatment interventions. As depicted in Figure 3, as expected, statistically significant rapid drop in body weight was observed for mice treated with 40 mg/kg/day of Orlistat for the first two weeks of treatment period followed by a moderate body weight gain compared to vehicle treated HFD group. On the other hand, mice treated with 1.3 g/kg/day of UP601 showed very stable decrease in body weight throughout the duration of treatment. Compared to vehicle treated HFD group, body weight gains for mice treated with UP601 were significantly lower as of week 1 of treatment and these differences remained statistically significant for the rest of treatment duration (Figure 3). The changes in body weight gains at week 7 were found to be 2.42 ± 1.5, 6.59 ± 1.5, 2.35 ± 3.5, and −3.39 ± 2.7 grams for normal control diet, HFD, Orlistat, and UP61 treated animals, respectively (Figure 3).

### 3.3. Effect of UP601 on Calorie Intake in the DIO Model

**Figure 4:**
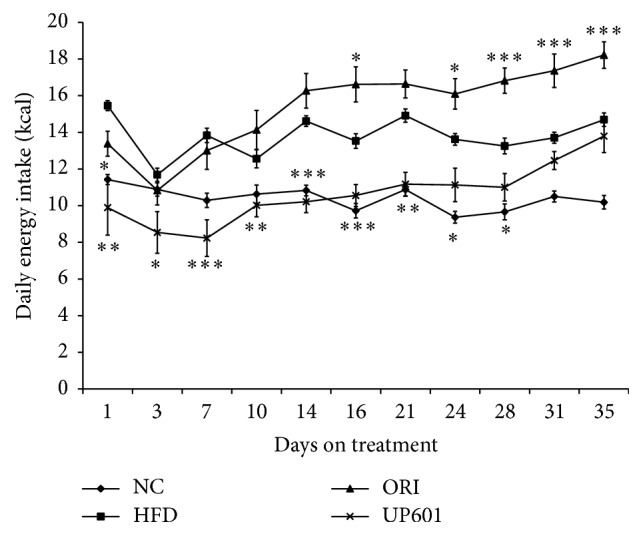
Daily calorie intake by obese mice treated with oral doses of UP601 at 1.3 g/kg in the DIO model. ^*∗∗∗*^ *P* ≤ 0.0001; ^*∗∗*^ *P* ≤ 0.001; ^*∗*^ *P* ≤ 0.05.

To better understand the possible mechanism(s) underlying homeostatic control mechanisms of energy intake to expenditure and the subsequent weight gain, feed consumption was monitored twice a week for the course of treatment. As described in Figure 4, obese mice treated with Orlistat consumed similar amount of calorie intake for the first week of treatment followed by a significant increase in calorie intake comparable to that of the HFD group which lasted for the rest of treatment duration. In contrast, mice treated with UP601 showed statistically significant 36%, 26.9%, and 40.5% decrease in calorie intake on days 1, 3, and 7, respectively, after treatment and remained lower calorie intake for the duration of treatment period compared to vehicle treated HFD group (Figure 4).

### 3.4. Effect of UP601 on Liver and Lipid Biomarkers in the DIO Model

Dyslipidemia is the major component of metabolic disorder that could occur as a result of consumption of high-fat diet where the levels of LDL-cholesterol with direct correlation of cardiovascular risk. To evaluate this association and impact of daily oral administration of UP601 to mice, serum samples were collected and levels of liver enzymes as well as lipid biomarkers were determined. As illustrated in Table 1, the composition restored altered metabolic disturbances as demonstrated by restoration of serum liver enzymes and lipid profiles towards near normal levels. 59% reduction in LDL was observed for mice treated with UP601 at a dose of 1.3 g/kg compared to vehicle treated HFD group. Similarly, 18.6% reduction in total cholesterol was observed for mice treated with the composite at oral doses of 1.3 g/kg for 7 weeks. Significantly low levels of liver enzymes (ALT = 60.1% decrease and AST = 35.2% decrease) were also observed for mice treated with UP601 when compared to vehicle treated HFD mice. Mice treated with Orlistat showed 69.1%, 24.2%, 25.7%, and 52.4% reductions in ALT, AST, total cholesterol, and LDL, respectively, compared to vehicle treated HFD (Table 1).

### 3.5. Effect of UP601 on Body Composition and Relative Organ Weight in the DIO Model

**Figure 5:**
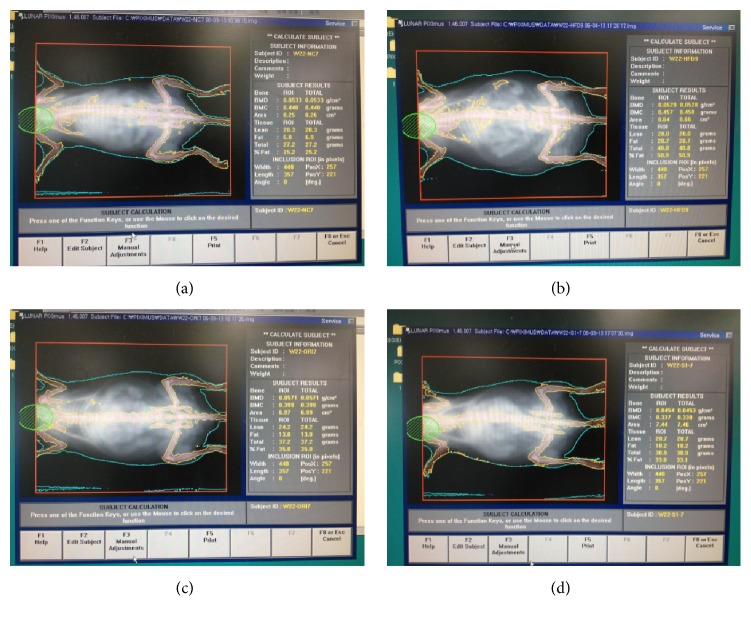
DEXA scan images for mice in the HFD-induced obesity group. (a) Normal control; (b) HFD + vehicle; (c) HFD + ORI; (d) HFD + 1.3 g UP601.

**Figure 6:**
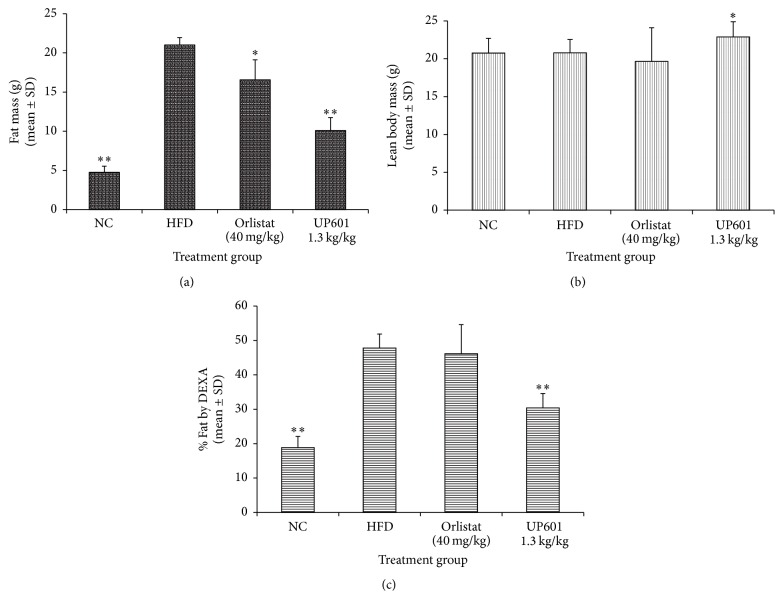
DEXA scan measurements for lean body and fat mass. (a) Fat mass, (b) lean body mass, and (c) percent fat relative to body weight. ^*∗∗*^ *P* ≤ 0.0001; ^*∗*^ *P* ≤ 0.001.

Dual energy X-ray absorptiometry (DEXA) scan was performed to determine the lean body and fat mass distribution of sedated mice (Figures 5 and 6). After 7 weeks of daily oral treatment, statistically significant increase in lean body mass and decreases in fat mass were observed for mice treated with UP601 compared to HFD. The positive control Orlistat showed statistically significant decrease in fat mass compared to vehicle treated HFD mice (Figure 6). In relative to their body weight, the percentage fat was found to be 18.9%, 47.8%, 46.1%, and 30.4% for mice treated with normal control, HFD, Orlistat, and UP601, respectively (Figure 6). Mice treated with UP601 showed 36.4% relative decrease in body fat mass compared to the HFD group. In agreement with the DEXA scan data, a decrease in wet tissue visceral fat weight was observed for mice treated with UP601 compared to vehicle treated HFD mice where most of the reductions were contributed by low mass in the mesenteric and perirenal fat (Table 2). No difference in liver weight was observed for mice treated with UP601 and in the regular diet group when compared to the HFD. Mice treated with Orlistat showed a statistically significant decrease in the level of fat mass at perirenal and mesenteric fat; however, the total fat mass was not different from vehicle treated HFD mice (Table 2). On the other hand, statistically significant decrease in relative liver weight was observed for mice treated with Orlistat when compared to HFD group (Table 2).

### 3.6. Effect of UP601 on Liver Histopathology in the DIO Model

**Figure 7:**
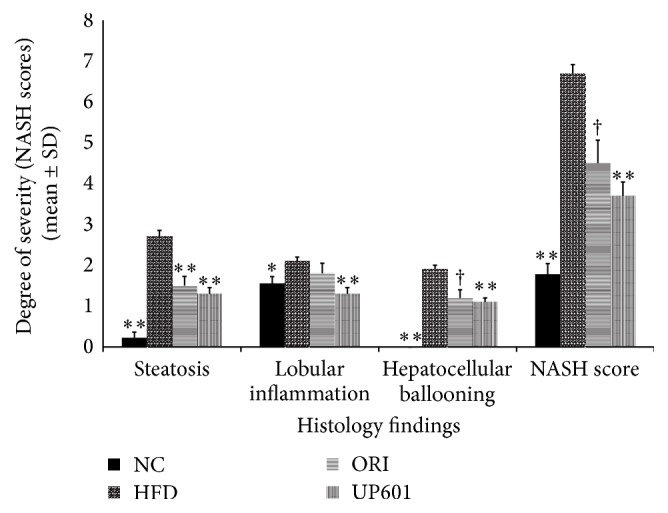
Nonalcoholic steatohepatitis scores for mice in the HFD study group treated with Orlistat and UP601 at doses of 1.3 g/kg. ^*∗∗*^ *P* ≤ 0.0001; ^†^ *P* ≤ 0.001; ^*∗*^ *P* ≤ 0.05.

**Figure 8:**
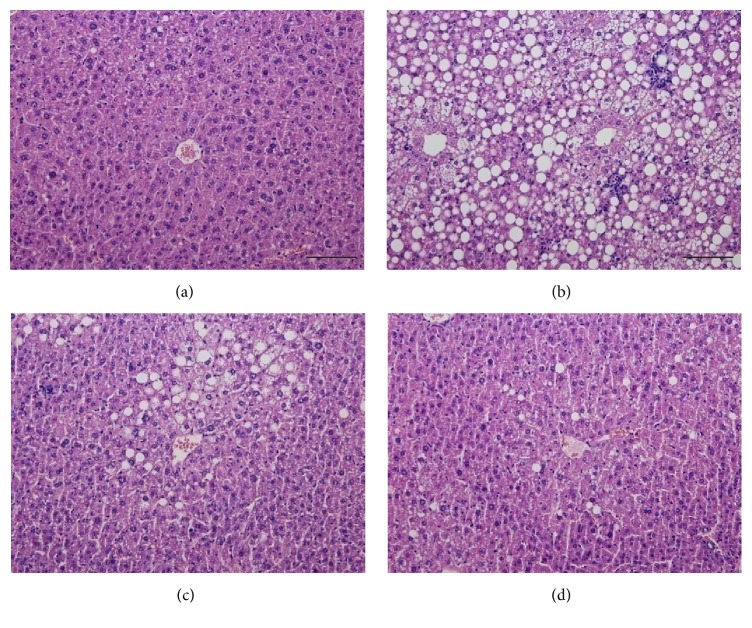
H&E staining of liver tissue. (a) Normal control; (b) HFD (60% fat calorie diet group); (c) HFD + Orlistat treated (40 mg/kg); (d) HFD + 1.3 g/kg UP601 treated. Magnification is ×200 and the bar means 100 *μ*m.

A rapid onset of lipid accumulation in the liver with both macro- and microvacuolation is a common phenomenon when mice are fed with HFD, which becomes more severely untreated with increasing duration. Histological analysis of liver sections from the HFD groups of mice demonstrated enlargement of hepatocytes and steatosis represented by the vacuolation in hepatocytes of the vehicle treated HFD mice in comparison with those in the normal control mice. As the histopathology data showed, after 7 weeks of daily oral treatment, significantly improved NASH scores in steatosis, lobular inflammation, and hepatocellular ballooning were observed for mice treated with UP601 or Orlistat compared to vehicle treated HFD group (Figures 7 and 8).

### 3.7. Effect of UP601 on the Metabolic Biomarkers in the DIO Model

**Figure 9:**
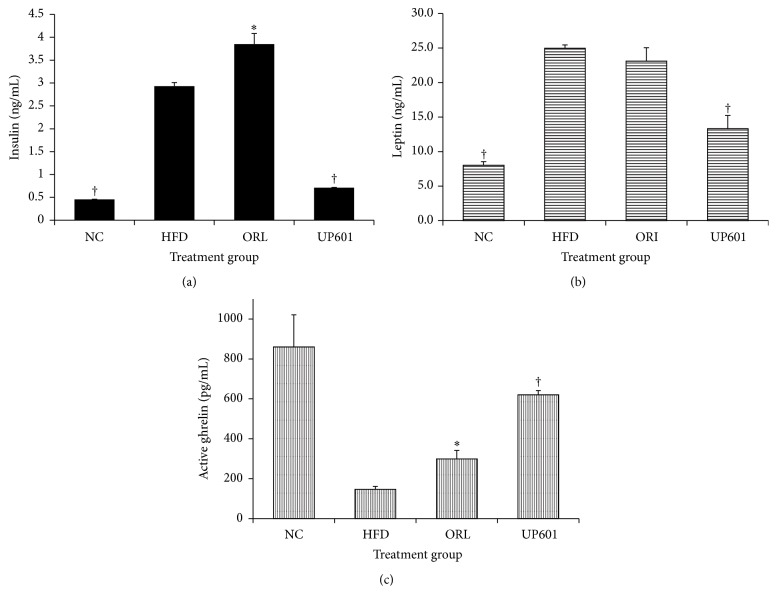
Insulin (a), leptin (b), and ghrelin level (c) of obese mice treated with UP601 at oral doses of 1.3 g/kg for 7 weeks. ^†^ *P* ≤ 0.0001; ^*∗*^ *P* ≤ 0.05.

Serum levels of insulin, leptin, and ghrelin were measured for mice treated with UP601 at oral dose of 1.3 g/kg for 7 weeks. As depicted in Figure 9, obese mice treated with vehicle showed almost 6-fold and 3-fold increase in serum insulin and leptin levels, respectively, and 6-fold decrease in active ghrelin level indicating the presence of altered metabolism. Mice in the UP601 treatment group showed 75.9% decrease in insulin, 46.8% decrease in leptin, and 4.2-fold increase in ghrelin level compared to the vehicle treated obese mice. The reference compound Orlistat showed 34.5% increase in insulin, 7.6% decrease in leptin, and 2-fold increase in ghrelin compared to the vehicle treated obese mice.

## 4. Discussion

Global efforts to treat the obesity pandemic have not been very successful. While it is a preventable disorder, obesity continues to be one of the biggest worldwide challenges in the 21st century, with more than 1.9 billion of adults considered being overweight of whom over 600 million were obese in the year 2014 [1]. The trend is expected to grow if undressed. The current antiobesity pharmaceutical drugs, Orlistat (Xenical®/Alli; gastric and pancreatic lipase inhibitor, FDA approved 1999) [24], lorcaserin (Belviq®; FDA approved 2012, a potent and selective agonist of 5-HT2C receptor marketed for appetite suppression) [25], and Qysmia (phentermine, a sympathomimetic amine anorectic, and topiramate extended release, an antiepileptic drug, FDA approved 2012) [26] cannot be the magical “cure all” solution and are not without associated side effects. As a result, novel pharmaceutical or nutritional based therapeutic agents are urgently required. In this respect, traditional medicines may offer various medical foods or dietary supplements for reducing appetite and hence body weight that warrant further considerations based on their risk-benefit assessment.

In the present study, the biochemical and biological activity of a well-defined composition, UP601, from three historically very well-known plant extracts (*Morus alba*,* Yerba mate*, and* Magnolia officinalis*) were addressed in fully established animal obesity models that mimic human metabolic disturbances frequently exhibited as a result of consumption of western diets. The appetite suppression effects of the composition were also assessed.

As evidenced in multiple animal models, these plant extracts presumed to contain bioactive compounds that possess metabolic disease-modifying activities beneficial to the effects observed in the present study. For example, extracts from* Magnolia* have shown decrease in fasting blood glucose and plasma insulin as well as diabetic nephropathy prevention in type 2 diabetic Goto-Kakizaki rats [27], attenuate adipogenic differentiation in 3T3-L1 adipocytes [28], stimulate glucose uptake in insulin-sensitive and insulin-resistant murine and human adipocytes using the insulin signaling pathway [29], and control elevated stress-related cortisol level [30] in human. The beneficial moderation in appetite suppression, body weight, and metabolic markers observed in the current study could partially be explained by the inherent activities of* Magnolia* extract contributed to the composition as described in this summary.

Significant studies have also demonstrated that* Yerba mate* extracts are rich in polyphenols, xanthines, purine alkaloids, and flavonoids to alleviate weight gain and to improve plasma glucose and lipid profiles in animals. The impacts of* Yerba mate* on obesity and insulin resistance in animals and humans in association with its potential mechanisms of actions have been well reviewed by Gambero and Ribeiro [12, 31] who have reported marked attenuation of weight gain, adiposity, a decrease in epididymal fat-pad weight, and restoration of the serum levels of cholesterol, triglycerides, LDL-cholesterol, and glucose in HFD-induced obese mice treated orally with mate extract for 8 weeks at a dose of 1 g/kg. These results were identical to what was observed in our study. Additionally, significantly lowered body weight, visceral fat-pad weights, blood and hepatic lipid, glucose, insulin, and leptin levels were observed in HFD rats treated with* Yerba mate* extract formulated diet for 60 days [15]. Here, again, the composition UP601 might have been assumed to adapt the suggested mechanisms of actions and activities of* Yerba mate* as shown by data depicted in the current study.

Multiple studies have also reported the merit of* Morus* extracts in metabolic disorders like diabetes and obesity. Hypoglycemic activities in diabetes patients [32, 33] improving hyperglycemia and associated complications in the diabetic rats [34], decreasing body weight and adiposity, regulating hepatic lipid accumulation in diet induced obese mice [16], and decreasing expression of white muscle adipocytokines in db/db mice [18] and blood glucose level in alloxan diabetic mice [19] were some of the beneficial effects documented for this plant. Previously it has also been reported that* Morus* extract increases adiponectin level of adipocytes [20], increases glucose uptake and GLUT4 translocation in adipocytes [20], inhibits *α*-glucosidase activity [21], and inhibits human and rat intestinal disaccharidase [35]. In support of the other two components of the composition,* Morus extract* also reenforces the potential of UP601 to yield a boosted efficacy in weight management as a result of collective mechanism of actions contributed by all the three components.

Therefore, it is possible to hypothesize that the composition to have diversified components and characteristics with compounding effects in moderating metabolic disorders signifying the merit of their combination for weight management is given the above individual reports of each component of the composition. The hypothesis was tested and beneficial weight management activities were confirmed by an acute food intake study in rats and HFD-induced mouse obesity models. UP601 effectively attenuated calorie intake, body weight gain, and counteracts metabolic disorders, especially obesity-associated liver damage, induced by a HFD in mice.

The composition UP601 produced marked acute hypophagia in rats. Statistically significant reductions in acute food intake were observed for the first 4 hours after food provision for rats treated with UP601 when consumption was monitored hourly. Similarly, the cumulative food intake data showed statistically significant reductions in food intake for 24 hours except for the low-dose 250 mg/kg UP601 group which did not show the significance at the 6-hour time point. In agreement with our data, for example, the pharmaceutical drug lorcaserin, a selective human 5-hydroxytryptamine-2C (5-HT)_2C_ agonist resulted in reduced acute food intake in rats administered orally at doses of 3–24 mg/kg. At the highest dose tested, 80, 65, 55, and 25% inhibitions in cumulative food intakes were observed at 2, 4, 6, and 22 hours, respectively, after the provision of food [36]. For a head to head comparison, 82, 75, 44, and 31% reductions in cumulative food intake were observed at 2, 4, 6, and 24 hours, respectively, after the provision of food for rats treated with UP601 at an oral dose of 230 mg/kg. In a similar study when potato protease inhibitory concentrate (PPIC) was tested on normal rats, a significant reduction in food intake was seen in the 1st (41.2%) and 2nd (42.6%) hours following oral administration of 100 mg/kg to 6 h fasted rats in the early dark phase presumed through increasing circulating CCK levels through a trypsin-dependent mechanism. A residual nonsignificant 6.9% decrease in food intake was also observed at the 24-hour time point [37].

In addition to the acute feed intake observations, data depicted here show that chronic UP601 oral administration reduces food intake and weight gain in C57BL/6J obese mice fed HFD, demonstrating the consistent efficacy of UP601. In the repeated dose study, 21.5% reductions in body weight gain were observed for obese mice treated with UP601 at doses of 1.3 g/kg when compared to vehicle treated HFD group at week 7. Compared to week 0 (baseline or treatment start), the composition resulted in moderate and progressive decrease in body weight for the duration of the treatment with 8.2% reduction at the end of study. In contrast, the HFD group gained 15.9% and the Orlistat group gained 5.7% of their treatment start initial body weight. In agreement with the acute feed intake data, here, there was up to 40.5% decrease in calorie intake for the first week of treatment of UP601 and remained lower for the duration of treatment compared to the vehicle treated HFD group reflecting the appetite suppression activity of the composition. It was noticed that the marked acute hypophagia observed in the acute feed intake study tends to attenuate over the repeated administration. Consistent with our findings, rimonabant administered orally to DIO mice at oral doses of 10 mg/kg/day for 5 weeks resulted in transient reductions in daily calorie intake (48% for the first week compared to vehicle treated HFD) and was accompanied by marked reductions in body weight, 20% lighter than the vehicle treated obese mice [38].

Insulin resistance, the fundamental etiology of type 2 diabetes, is one of the primary complications of obesity [39]. Untreated, it can result in deleterious consequences characterized under metabolic syndrome that includes hypertension, hyperlipidemia, and atherosclerosis [40]. Patients with chronic metabolic imbalances experience high level of insulin. This phenomenon was observed in the vehicle treated HFD group in the current study. However, the surge of insulin was reduced to the level of the normal control mice as a result of the 7-week oral treatment of UP601. Similarly, statistically nonsignificant increases in serum glucose level were observed for the HFD mice treated with vehicle at the end of the study. In contrast, mice treated with the composition showed a similar level of blood glucose to that of the regular diet fed mice. There were 14.4% and 13.6% decreases in blood glucose levels for the regular diet fed normal control mice and UP601 treated mice, respectively, compared to the HFD. These changes were not statistically significant compared to HFD. The positive control Orlistat resulted in 9.6% reductions in the blood glucose level compared to HFD. The C57BL/6J mice are known to become obese when fed with the HFD; however, they are prone to produce mild to moderate hyperglycemia [41].

The anorexigenic hormone, leptin, and the orexigenic hormone, ghrelin, are two hormones that have been recognized to have a major influence on energy balance and obesity. While leptin mediates the long-term regulation of energy balance by suppressing food intake, ghrelin seems to play a role in meal initiation. Clinically, obese subjects have increased level of circulating leptin and decreased level of ghrelin [42]. Again, these phenomena were observed in the vehicle treated HFD group in the current study. In contrast, these abnormal levels of leptin and ghrelin were reversed significantly towards the normal level due to UP601 treatment. These findings further give strong support to the efficacy of UP601 in moderation of calorie intake and metabolic disorders.

The beneficial metabolic management effects observed with UP601 were very comparable to or better than those found with the Food and Drug Administration that approved pancreatic lipase inhibitor used as reference compound, Orlistat. We have found, however, that treatment with UP601 did not produce associated side effects usually exhibited in this class of drugs such as diarrhea, fast drop in body weight, increase in calorie intake, and steatorrhea.

As evidenced by the liver histology and serum biochemical data, UP601 treatment prevented hepatic steatosis induced by the HFD diet. Treatments of HFD diet-induce obese mice with UP601 significantly reduced total cholesterol and LDL levels which agree with the findings for* Morus*,* Magnolia*, and* Yerba mate* as mentioned in the body of the manuscripts above. Thus, UP601 appears to possess additional beneficial effects in regulation of dyslipidemia. Furthermore, mice treated with UP601 showed significantly improved NASH scores in steatosis, lobular inflammation, and hepatocellular ballooning without affecting the liver enzymes (AST and ALT) suggesting that the composition may have an indication in nonalcoholic fatty liver disease without associated adverse effects. Therefore, the data depicted in the current study solidifies the merit of combining these plant extracts for a better outcome targeting multiple pathways that are affected in various aspects of metabolic disorders.

Mounting evidences have been reported to show the strong association of abdominal obesity with metabolic disorders in humans [43]. It was found that the 7-week oral UP601 treatment significantly decreased total fat, percent body fat, and visceral fat weights. As depicted in the DEXA scan data, obese mice treated orally with UP601 showed a decrease in fat mass with an increase in lean body mass, which magnifies the decrease in body weight gain observed in these mice mainly due to the result of reduced visceral fat accumulation. In agreement with this occurrence, the relative organ weight data also demonstrated statistically significant lower degree of perirenal and mesenteric fat deposits where the largest decrease occurring in the mesenteric depots was 59.3% less in UP601 group compared to vehicle treated HFD. Comparable to our data, the weight loss observed in DIO mice treated with rimonabant for 5 weeks at oral dose of 10 mg/kg/day was associated with a depletion of fat stores reaching 50% due to the decrease in both the weight of abdominal fat pads and the total body fat content [38].

Animal models used in the discovery of novel treatments for obesity range from direct measures of food intake in lean rodents to long-term studies in animals exhibiting obesity due to the continuous access to highly palatable calorie-dense diets. Understanding the pathways implicated in appetite modulation is a necessity for the development of safe and effective weight loss products and hence further mechanism based elucidations are warranted for the composition. Regardless, the data depicted here suggest the appetite curving ability of UP601 in both the lean and obese rodent models.

Emphasizing obesity as a consequence of the imbalance between energy intake and energy expenditure, components of the composition could have impacted the reduction of energy intake by appetite suppression (e.g.,* Yerba mate* and* Magnolia* extract), inhibition of carbohydrate absorption (e.g.,* Morus* extract), increase of energy expenditure (e.g.,* Yerba mate* extract), and modulation of fat (e.g.,* Morus*,* Yerba mate* and* Magnolia* extracts) for the promising results documented here. Acknowledging the intricate nature of obesity and its comorbidities, future treatment strategies may rely on the use of combination therapy of two or more active compounds to address the multiple pathways involved in obesity. In fact, the use of Qysmia, the cocktail of phentermine, a sympathomimetic amine anorectic drug and topiramate, an antiepileptic drug for weight loss could be considered as a good example for this strategy.

In summary, we have standardized and demonstrated a novel and efficacious composition from historically well-known plants,* Morus alba*,* Yerba mate*, and* Magnolia officinalis*. The present study shows UP601 to possess an appetite reducing activity as reflected by the reduced food intake both in the acute and in chronic models. Oral administration of UP601 to the mice for 7 weeks resulted in sustained reductions in body weight gain compared to vehicle. While preclinical to clinical data translation requires human clinical trial validation, the data depicted here and individual components safe historical usage suggest UP601 could potentially be considered as a natural alternative to suppress appetite, maintain healthy body weight, and manage metabolism.

## Figures and Tables

**Table 1:** Liver function and lipid profiling.

Group	ALT (U/L)	AST (U/L)	Glucose (mg/dL)	T-chol (mg/dL)	TG (mg/dL)	LDL-C (mg/dL)	HDL-C (mg/dL)
NC	12.3 ± 2.7^*∗∗*^	40.8 ± 4.3^*∗∗*^	232.7 ± 65.6	107.3 ± 2.5^*∗∗*^	24.3 ± 10.5	7.7 ± 1.2^*∗*^	49.2 ± 1.0^*∗∗*^
HFD	88.8 ± 24.7	86.0 ± 10.9	272.0 ± 34.4	194.3 ± 17.2	24.8 ± 8.3	10.5 ± 1.1	68.7 ± 5.1
ORI	27.4 ± 4.8^*∗*^	65.2 ± 21.6	245.8 ± 38.1	144.3 ± 36.0^*∗*^	45.8 ± 22.1	5.0 ± 0.8^*∗∗*^	66.1 ± 14.6
UP601	35.4 ± 7.2^*∗*^	55.7 ± 7.5^*∗∗*^	235.0 ± 92.4	158.2 ± 10.7^*∗∗*^	35.0 ± 18.2	4.3 ± 0.7^†^	70.1 ± 3.7

^*∗*^ *P* ≤ 0.05; ^*∗∗*^ *P* ≤ 0.0001; ^†^ *P* ≤ 0.001. *P* values versus HFD group.

**Table 2:** Relative organ weight for mice treated with UP601 in HFD-induced mouse obesity model.

Groups	Liver	Fat deposition				
		Epididymal	Retroperitoneal	Perirenal	Mesenteric	Total
NC	3.49 ± 0.61	2.38 ± 1.03	0.62 ± 0.33	0.33 ± 0.10^‡^	1.04 ± 0.29^‡^	4.37 ± 1.70^‡^
HFD	3.54 ± 0.81	4.94 ± 0.76	1.14 ± 0.16	1.31 ± 0.15	3.51 ± 0.48	10.90 ± 0.63
Orlistat (40 mg/kg)	2.61 ± 0.45^†^	5.78 ± 0.87	1.36 ± 0.25	0.98 ± 0.21^†^	2.54 ± 0.52^†^	10.66 ± 1.07
UP601 (1.3 g/kg)	3.14 ± 0.13	4.36 ± 0.53^*∗*^	1.44 ± 0.15	0.63 ± 0.09^‡^	1.43 ± 0.32^‡^	7.55 ± 0.82^‡^

^*∗*^ *P* ≤ 0.05; ^†^ *P* ≤ 0.001; ^‡^ *P* ≤ 0.0001. *P* values versus HFD group.

## References

[1] World Health Organization (2015). *Obesity and Overweight. Fact Sheet No 311*.

[2] Di Cesare M., Bentham J., Stevens G. A. (2016). Trends in adult body-mass index in 200 countries from 1975 to 2014: a pooled analysis of 1698 population-based measurement studies with 19.2 million participants. *The Lancet*.

[3] Haslam D. W., James W. P. T. (2005). Obesity. *The Lancet*.

[4] Li M.-F., Cheung B. M. Y. (2011). Rise and fall of anti-obesity drugs. *World Journal of Diabetes*.

[5] Swinburn B. A., Caterson I., Seidell J. C., James W. P. T. (2004). Diet, nutrition and the prevention of excess weight gain and obesity. *Public Health Nutrition*.

[6] Choi S.-S., Cha B.-Y., Lee Y.-S. (2009). Magnolol enhances adipocyte differentiation and glucose uptake in 3T3-L1 cells. *Life Sciences*.

[7] Kubota M. (1996). The therapeutic effect of saiboku-to on anxiety disorders and others. *J. Tradit. Sino-lpn. Med*.

[8] Kuribara H., Morita M., Ishige A. (1996). Investigation of the anxiolylic effect of the extracts derived from saiboku-to, an oriental herbal medicine, by an improved plus-maze test in mice. *Japanese Journal of Neuropsychopharmacology*.

[9] Ikarashi Y., Yuzurihara M., Sakakibara I., Nakai Y., Hattori N., Maruyama Y. (2001). Effects of the extract of the bark of *Magnolia obovata* and its biphenolic constituents magnolol and honokiol on histamine release from peritoneal mast cells in rats. *Planta Medica*.

[10] Ikarashi Y., Yuzurihara M. (2002). Potentiation by *saiboku-to* of diazepam-induced decreases in hippocampal and striatal acetylcholine release in rats. *Phytomedicine*.

[11] Kang Y. R., Lee H. Y., Kim J. H. (2012). Anti-obesity and anti-diabetic effects of Yerba Mate (*Ilex paraguariensis*) in C57BL/6J mice fed a high-fat diet. *Laboratory Animal Research*.

[12] Arçari D. P., Bartchewsky W., Dos Santos T. W. (2009). Antiobesity effects of yerba maté extract (ilex paraguariensis) in high-fat diet-induced obese mice. *Obesity*.

[13] Arçari D. P., Santos J. C., Gambero A., Ribeiro M. L. (2013). The in vitro and in vivo effects of yerba mate (Ilex paraguariensis) extract on adipogenesis. *Food Chemistry*.

[14] Martins F., Noso T. M., Porto V. B. (2010). Maté tea inhibits in vitro pancreatic lipase activity and has hypolipidemic effect on high-fat diet-induced obese mice. *Obesity*.

[15] Pang J., Choi Y., Park T. (2008). Ilex paraguariensis extract ameliorates obesity induced by high-fat diet: potential role of AMPK in the visceral adipose tissue. *Archives of Biochemistry and Biophysics*.

[16] Oh K.-S., Ryu S. Y., Lee S. (2009). Melanin-concentrating hormone-1 receptor antagonism and anti-obesity effects of ethanolic extract from *Morus alba* leaves in diet-induced obese mice. *Journal of Ethnopharmacology*.

[17] Tanabe K., Nakamura S., Omagari K., Oku T. (2011). Repeated ingestion of the leaf extract from *Morus alba* reduces insulin resistance in KK-Ay mice. *Nutrition Research*.

[18] Sugimoto M., Arai H., Tamura Y. (2009). Mulberry leaf ameliorates the expression profile of adipocytokines by inhibiting oxidative stress in white adipose tissue in db/db mice. *Atherosclerosis*.

[19] Zhang M., Chen M., Zhang H.-Q., Sun S., Xia B., Wu F.-H. (2009). In vivo hypoglycemic effects of phenolics from the root bark of Morus alba. *Fitoterapia*.

[20] Naowaboot J., Pannangpetch P., Kukongviriyapan V., Prawan A., Kukongviriyapan U., Itharat A. (2012). Mulberry leaf extract stimulates glucose uptake and GLUT4 translocation in rat adipocytes. *The American Journal of Chinese Medicine*.

[21] Tao Y., Zhang Y., Cheng Y., Wang Y. (2013). Rapid screening and identification of *α*-glucosidase inhibitors from mulberry leaves using enzyme-immobilized magnetic beads coupled with HPLC/MS and NMR. *Biomedical Chromatography*.

[22] Hariri N., Thibault L. (2010). High-fat diet-induced obesity in animal models. *Nutrition Research Reviews*.

[23] Kleiner D. E., Brunt E. M., Van Natta M. (2005). Design and validation of a histological scoring system for nonalcoholic fatty liver disease. *Hepatology*.

[24] Cheah J. S. (2000). Orlistat (Xenical) in the management of obesity. *Annals of the Academy of Medicine Singapore*.

[25] Fleming J. W., McClendon K. S., Riche D. M. (2013). New obesity agents: lorcaserin and phentermine/topiramate. *Annals of Pharmacotherapy*.

[26] Kelly E. M., Tungol A. A., Wesolowicz L. A. (2013). Formulary management of 2 new agents: lorcaserin and phentermine/topiramate for weight loss. *Journal of Managed Care Pharmacy*.

[27] Sohn E. J., Kim C.-S., Kim Y. S. (2007). Effects of magnolol (5,5′-diallyl-2,2′-dihydroxybiphenyl) on diabetic nephropathy in type 2 diabetic Goto-Kakizaki rats. *Life Sciences*.

[28] Kong C.-S., Lee J. I., Kim J.-A., Seo Y. (2011). *In vitro* evaluation on the antiobesity effect of lignans from the flower buds of *Magnolia denudata*. *Journal of Agricultural and Food Chemistry*.

[29] Alonso-Castro A. J., Zapata-Bustos R., Domínguez F., García-Carrancá A., Salazar-Olivo L. A. (2011). *Magnolia dealbata* Zucc and its active principles honokiol and magnolol stimulate glucose uptake in murine and human adipocytes using the insulin-signaling pathway. *Phytomedicine*.

[30] Garrison B., Hughes K. (2005). Relaxation during weight loss: relieving stress with an herbal combination. *Alternative and Complementary Therapies*.

[31] Gambero A., Ribeiro M. L. (2015). The positive effects of yerba maté (Ilex paraguariensis) in obesity. *Nutrients*.

[32] Andallu B., Suryakantham V., Lakshmi Srikanthi B., Kesava Reddy G. (2001). Effect of mulberry (*Morus indica* L.) therapy on plasma and erythrocyte membrane lipids in patients with type 2 diabetes. *Clinica Chimica Acta*.

[33] Murata K., Yatsunami K., Fukuda E. (2004). Antihyperglycemic effects of propolis mixed with mulberry leaf extract on patients with type 2 diabetes. *Alternative Therapies in Health and Medicine*.

[34] Andallu B., Varadacharyulu N. C. (2007). Gluconeogenic substrates and hepatic gluconeogenic enzymes in streptozotocin-diabetic rats: effect of mulberry (*Morus indica* L.) leaves. *Journal of Medicinal Food*.

[35] Oku T., Yamada M., Nakamura M., Sadamori N., Nakamura S. (2006). Inhibitory effects of extractives from leaves of Morus alba on human and rat small intestinal disaccharidase activity. *British Journal of Nutrition*.

[36] Thomsen W. J., Grottick A. J., Menzaghi F. (2008). Lorcaserin, a novel selective human 5-hydroxytryptamine_2C_ agonist: in vitro and in vivo pharmacological characterization. *Journal of Pharmacology and Experimental Therapeutics*.

[37] Komarnytsky S., Cook A., Raskin I. (2011). Potato protease inhibitors inhibit food intake and increase circulating cholecystokinin levels by a trypsin-dependent mechanism. *International Journal of Obesity*.

[38] Ravinet Trillou C., Arnone M., Delgorge C. (2003). Anti-obesity effect of SR141716, a CB1 receptor antagonist, in diet-induced obese mice. *American Journal of Physiology—Regulatory Integrative and Comparative Physiology*.

[39] Chang Y.-H., Chang D.-M., Lin K.-C., Shin S.-J., Lee Y.-J. (2011). Visfatin in overweight/obesity, type 2 diabetes mellitus, insulin resistance, metabolic syndrome and cardiovascular diseases: a meta-analysis and systemic review. *Diabetes/Metabolism Research and Reviews*.

[40] Grundy S. M. (2016). Metabolic syndrome update. *Trends in Cardiovascular Medicine*.

[41] Hoffler U., Hobbie K., Wilson R. (2009). Diet-induced obesity is associated with hyperleptinemia, hyperinsulinemia, hepatic steatosis, and glomerulopathy in C57Bl/6J mice. *Endocrine*.

[42] Lim H. H., Yang S. J., Kim Y., Lee M., Lim Y. (2013). Combined treatment of mulberry leaf and fruit extract ameliorates obesity-related inflammation and oxidative stress in high fat diet-induced obese mice. *Journal of Medicinal Food*.

[43] Balkau B., Deanfield J. E., Després J.-P. (2007). International day for the evaluation of abdominal obesity (IDEA): a study of waist circumference, cardiovascular disease, and diabetes mellitus in 168 000 primary care patients in 63 countries. *Circulation*.

